# (2,2-Dimethyl-1,3-dioxolan-4-yl)methyl 3-carboxy­propanoate

**DOI:** 10.1107/S1600536809015190

**Published:** 2009-05-07

**Authors:** Piotr Kuś, Marcin Rojkiewicz, Grzegorz Zięba, Monika Witoszek, Peter G. Jones

**Affiliations:** a Department of Chemistry, University of Silesia, 9 Szkolna Street, 40-006 Katowice, Poland; b Institut für Anorganische und Analytische Chemie, Technische Universität Braunschweig, Postfach 3329, 38023 Braunschweig, Germany

## Abstract

In the title compound, C_10_H_16_O_6_, the five-membered ring has an envelope conformation. The packing involves hydrogen-bonded carboxylic acid inversion dimers and three C—H⋯O inter­actions.

## Related literature

For related literature, see: Osanai *et al.* (1997 ▶); Scriba (1993 ▶, 1995 ▶). The structure of a related derivative is reported in the preceeding paper, see: Kuś *et al.* (2009 ▶). 
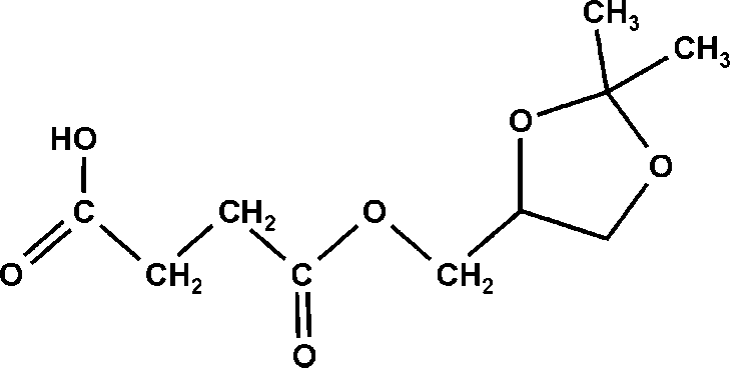


## Experimental

### 

#### Crystal data

C_10_H_16_O_6_
*M* *_r_* = 232.23
Monoclinic, 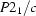

*a* = 20.7650 (12) Å
*b* = 5.7007 (3) Å
*c* = 9.6964 (7) Å
β = 98.658 (5)°
*V* = 1134.73 (12) Å^3^
*Z* = 4
Cu *K*α radiation
μ = 0.96 mm^−1^
*T* = 100 K
0.2 × 0.1 × 0.1 mm

#### Data collection

Oxford Diffraction Xcalibur diffractometer with an Atlas (Nova) detector
Absorption correction: multi-scan (*CrysAlis RED*; Oxford Diffraction, 2008 ▶) *T*_min_ = 0.880, *T*_max_ = 1.000 (expected range = 0.799–0.908)
10581 measured reflections
2304 independent reflections
2170 reflections with *I* > 2σ(*I*)
*R*_int_ = 0.028

#### Refinement

*R*[*F* ^2^ > 2σ(*F* ^2^)] = 0.051
*wR*(*F* ^2^) = 0.125
*S* = 1.14
2304 reflections
156 parameters
4 restraints
H atoms treated by a mixture of independent and constrained refinement
Δρ_max_ = 0.27 e Å^−3^
Δρ_min_ = −0.25 e Å^−3^

### 

Data collection: *CrysAlis CCD* (Oxford Diffraction, 2008 ▶); cell refinement: *CrysAlis RED* (Oxford Diffraction, 2008 ▶); data reduction: *CrysAlis RED*; program(s) used to solve structure: *SHELXS97* (Sheldrick, 2008 ▶); program(s) used to refine structure: *SHELXL97* (Sheldrick, 2008 ▶); molecular graphics: *XP* (Siemens, 1994 ▶); software used to prepare material for publication: *SHELXL97*.

## Supplementary Material

Crystal structure: contains datablocks I, global. DOI: 10.1107/S1600536809015190/bt2936sup1.cif

Structure factors: contains datablocks I. DOI: 10.1107/S1600536809015190/bt2936Isup2.hkl

Additional supplementary materials:  crystallographic information; 3D view; checkCIF report

## Figures and Tables

**Table 1:** Hydrogen-bond geometry (Å, °)

*D*—H⋯*A*	*D*—H	H⋯*A*	*D*⋯*A*	*D*—H⋯*A*
O1—H01⋯O2^i^	0.80 (3)	1.86 (3)	2.6582 (19)	175 (3)
C3—H3*A*⋯O3^ii^	0.99	2.36	3.211 (2)	144
C2—H2*B*⋯O4^iii^	0.99	2.57	3.489 (2)	155
C7—H7*B*⋯O5^iv^	0.99	2.60	3.506 (3)	152

Symmetry codes: (i) 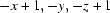; (ii) 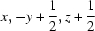; (iii) 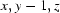; (iv) 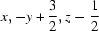.

**Fig. 1.**
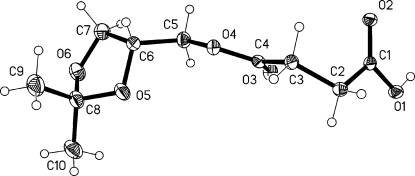
The title compound in the crystal structure. Displacement ellipsoids represent 50% probability levels.

**Fig. 2.**
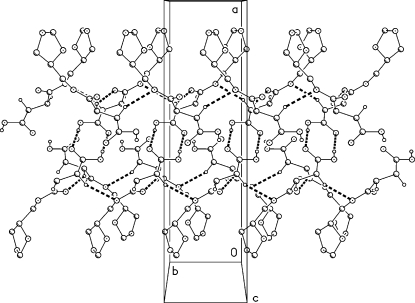
Packing diagram of the title compound. H atoms not involved in H bonding (thick dashed lines) are omitted for clarity. The interaction H7B···O5, which links the five-membered rings parallel to the c axis (the view direction), is not shown.

## supplementary crystallographic information

### Comment

Isopropylidene groups are often used as protecting or activating units in polyhydroxyalkyl compounds used for synthesis of sugar-like derivatives; for a brief introduction and the structure of a related derivative, see the accompanying paper (Kuś *et al.*, 2009).

Hemi-esters of succinic acid are often used for the synthesis of amphiphilic compounds with well organized structure (Osanai *et al.*, 1997). Non-symmetrical esters of succinic acid have been used for the synthesis of prodrugs that release the corresponding drugs very slowly; *e.g.* steroid drugs (Scriba, 1995) or Phenytoin (Scriba, 1993). Solketal (*D*,*L*-isopropylideneglycerol, Aldrich) was used for the synthesis of compound 1.

The molecule of compound 1 is shown in Fig. 1. Bond lengths and angles may be regarded as normal. The chain C2 through to C7 has an approximately extended conformation (absolute torsion angles between 158 and 174°). The five-membered ring displays an envelope conformation, with local mirror symmetry about C8 and the midpoint of C6—C7.

The molecular packing (Fig. 2) is dominated by the formation of the well known carboxylic acid dimers *via* classical hydrogen bonding. Three further contacts, of the type C—H···O, link the molecules to a three-dimensional pattern.

### Experimental

Compound 1 was obtained from solketal and succinic anhydride as described by Scriba (1993). Slow crystallization from petroleum ether gave crystals suitable for X-ray analysis. The analytical and spectroscopic data are consistent with the literature. M.p. 60° C. ^1^H NMR (CDCl3, 400 MHz): δ 4.32 (q, 1H), 4.21–4.05 (m, 3H), 3.75–3.72 (dd, 1H), 2.67 (t, 4H), 1.43 (s, 3H), 1.36 (s, 3H). ^13^C NMR (100 MHz): δ 207.34, 172.10, 110.03, 73.62, 66.39, 65.13, 31.03, 28.97, 26.78, 25.46. MS (ESI): m/*z* (%) = 231 (100) [M—H]^-^. IR: C═O at 1724, 1711 and 1694 cm^-1^ (*s*), C—O at 1234 cm^-1^ (*m*), 1,3-dioxalone at 975 cm^-1^ (*s*).

### Refinement

The OH hydrogen was refined freely. Methyl H atoms were identified in difference syntheses and refined as idealized rigid groups (C—H 0.98 Å, H—C—H 109.5°) allowed to rotate but not tip. Other H atoms were included at calculated positions and refined using a riding model, with fixed C—H bond lengths of 0.95 Å (CH, aromatic), 0.99 Å (CH_2_) and 1.00 Å (CH, *sp*^3^); *U*_iso_(H) values were fixed at 1.2*U*_eq_ of the parent C atom (1.2*U*_eq_ for methyl H).

The atom C6 is disordered over two sites with occupancy ratio 0.9:0.1, corresponding to a second conformation of the five-membered ring. An appropriate set of similarity restraints was used to ensure stability of refinement.

### Crystal data

**Table:**

C_10_H_16_O_6_	*D*_x_ = 1.359 Mg m^−^^3^
*M**_r_* = 232.23	Melting point: 333 K
Monoclinic, *P*2_1_/*c*	Cu *K*α radiation, λ = 1.54184 Å
*a* = 20.7650 (12) Å	Cell parameters from 7029 reflections
*b* = 5.7007 (3) Å	θ = 4.3–75.7°
*c* = 9.6964 (7) Å	µ = 0.96 mm^−^^1^
β = 98.658 (5)°	*T* = 100 K
*V* = 1134.73 (12) Å^3^	Block, colourless
*Z* = 4	0.2 × 0.1 × 0.1 mm
*F*(000) = 496	

### Data collection

**Table:**

Oxford Diffraction Xcalibur diffractometer with an Atlas (Nova) detector	2304 independent reflections
Radiation source: Nova (Cu) X-ray Source	2170 reflections with *I* > 2σ(*I*)
mirror	*R*_int_ = 0.028
Detector resolution: 10.3543 pixels mm^-1^	θ_max_ = 74.5°, θ_min_ = 4.3°
ω scans	*h* = −25→25
Absorption correction: multi-scan (*CrysAlis RED*; Oxford Diffraction, 2008)	*k* = −7→6
*T*_min_ = 0.880, *T*_max_ = 1.000	*l* = −10→12
10581 measured reflections	

### Refinement

**Table:**

Refinement on *F*^2^	Primary atom site location: structure-invariant direct methods
Least-squares matrix: full	Secondary atom site location: difference Fourier map
*R*[*F*^2^ > 2σ(*F*^2^)] = 0.051	Hydrogen site location: inferred from neighbouring sites
*wR*(*F*^2^) = 0.125	H atoms treated by a mixture of independent and constrained refinement
*S* = 1.14	*w* = 1/[σ^2^(*F*_o_^2^) + (0.0399*P*)^2^ + 1.0966*P*] where *P* = (*F*_o_^2^ + 2*F*_c_^2^)/3
2304 reflections	(Δ/σ)_max_ = 0.015
156 parameters	Δρ_max_ = 0.27 e Å^−^^3^
4 restraints	Δρ_min_ = −0.25 e Å^−^^3^

### Special details

**Table:**

Geometry. All e.s.d.'s (except the e.s.d. in the dihedral angle between two l.s. planes) are estimated using the full covariance matrix. The cell e.s.d.'s are taken into account individually in the estimation of e.s.d.'s in distances, angles and torsion angles; correlations between e.s.d.'s in cell parameters are only used when they are defined by crystal symmetry. An approximate (isotropic) treatment of cell e.s.d.'s is used for estimating e.s.d.'s involving l.s. planes.
Refinement. Refinement of *F*^2^ against ALL reflections. The weighted *R*-factor *wR* and goodness of fit *S* are based on *F*^2^, conventional *R*-factors *R* are based on *F*, with *F* set to zero for negative *F*^2^. The threshold expression of *F*^2^ > σ(*F*^2^) is used only for calculating *R*-factors(gt) *etc*. and is not relevant to the choice of reflections for refinement. *R*-factors based on *F*^2^ are statistically about twice as large as those based on *F*, and *R*- factors based on ALL data will be even larger.

### Fractional atomic coordinates and isotropic or equivalent isotropic displacement parameters (Å^2^)

**Table:**

	*x*	*y*	*z*	*U*_iso_*/*U*_eq_	Occ. (<1)
O1	0.44071 (7)	−0.1502 (2)	0.58989 (15)	0.0287 (3)	
H01	0.4643 (14)	−0.164 (5)	0.533 (3)	0.047 (8)*	
O2	0.47778 (6)	0.2189 (2)	0.59298 (14)	0.0278 (3)	
C1	0.44377 (9)	0.0675 (3)	0.63543 (19)	0.0243 (4)	
C2	0.40364 (9)	0.1107 (3)	0.7496 (2)	0.0265 (4)	
H2A	0.4299	0.0701	0.8403	0.032*	
H2B	0.3653	0.0055	0.7356	0.032*	
C3	0.38031 (9)	0.3632 (3)	0.75537 (19)	0.0258 (4)	
H3A	0.3599	0.3849	0.8405	0.031*	
H3B	0.4183	0.4697	0.7620	0.031*	
C4	0.33223 (9)	0.4289 (3)	0.6303 (2)	0.0258 (4)	
O3	0.32078 (7)	0.3156 (3)	0.52376 (14)	0.0340 (4)	
O4	0.30257 (6)	0.6315 (2)	0.65113 (14)	0.0295 (3)	
C5	0.26115 (10)	0.7325 (4)	0.5323 (2)	0.0339 (5)	
H5A	0.2372	0.6073	0.4752	0.041*	0.893 (8)
H5B	0.2877	0.8205	0.4731	0.041*	0.893 (8)
H5C	0.2798	0.8886	0.5170	0.041*	0.107 (8)
H5D	0.2675	0.6354	0.4508	0.041*	0.107 (8)
O5	0.16891 (7)	0.7592 (3)	0.65090 (18)	0.0426 (4)	
O6	0.10733 (7)	0.9607 (3)	0.48082 (18)	0.0453 (4)	
C6	0.21452 (11)	0.8932 (4)	0.5879 (3)	0.0318 (7)	0.893 (8)
H6	0.2385	1.0041	0.6573	0.038*	0.893 (8)
C7	0.17233 (11)	1.0302 (5)	0.4709 (3)	0.0459 (6)	
H7A	0.1779	1.2014	0.4852	0.055*	0.893 (8)
H7B	0.1839	0.9886	0.3786	0.055*	0.893 (8)
H7C	0.1896	1.1553	0.5371	0.055*	0.107 (8)
H7D	0.1801	1.0664	0.3749	0.055*	0.107 (8)
C6'	0.1934 (9)	0.766 (4)	0.523 (2)	0.069 (10)*	0.107 (8)
H6'	0.1699	0.6485	0.4572	0.083*	0.107 (8)
C8	0.10751 (10)	0.8735 (4)	0.6185 (2)	0.0388 (5)	
C9	0.10142 (14)	1.0693 (5)	0.7197 (3)	0.0568 (7)	
H9A	0.1050	1.0054	0.8144	0.085*	
H9B	0.0590	1.1461	0.6952	0.085*	
H9C	0.1362	1.1841	0.7156	0.085*	
C10	0.05421 (12)	0.6939 (5)	0.6160 (3)	0.0532 (7)	
H10A	0.0609	0.5680	0.5508	0.080*	
H10B	0.0119	0.7686	0.5861	0.080*	
H10C	0.0552	0.6282	0.7097	0.080*	

### Atomic displacement parameters (Å^2^)

**Table:**

	*U*^11^	*U*^22^	*U*^33^	*U*^12^	*U*^13^	*U*^23^
O1	0.0314 (7)	0.0253 (7)	0.0316 (7)	−0.0017 (6)	0.0123 (6)	−0.0010 (6)
O2	0.0279 (7)	0.0250 (7)	0.0322 (7)	−0.0018 (5)	0.0100 (5)	−0.0003 (6)
C1	0.0223 (8)	0.0241 (9)	0.0256 (9)	0.0016 (7)	0.0007 (7)	0.0022 (7)
C2	0.0272 (9)	0.0277 (10)	0.0254 (9)	−0.0004 (7)	0.0062 (7)	0.0022 (8)
C3	0.0266 (9)	0.0265 (10)	0.0247 (9)	0.0005 (7)	0.0055 (7)	−0.0011 (7)
C4	0.0233 (9)	0.0273 (10)	0.0285 (9)	−0.0009 (7)	0.0097 (7)	0.0007 (8)
O3	0.0374 (8)	0.0391 (8)	0.0256 (7)	0.0069 (6)	0.0048 (6)	−0.0039 (6)
O4	0.0263 (7)	0.0284 (7)	0.0337 (7)	0.0036 (6)	0.0043 (5)	0.0010 (6)
C5	0.0313 (10)	0.0355 (11)	0.0351 (11)	0.0064 (9)	0.0060 (8)	0.0049 (9)
O5	0.0274 (7)	0.0484 (10)	0.0538 (10)	0.0046 (7)	0.0119 (7)	0.0075 (8)
O6	0.0294 (8)	0.0545 (11)	0.0509 (10)	0.0047 (7)	0.0020 (7)	0.0003 (8)
C6	0.0259 (12)	0.0271 (12)	0.0427 (14)	−0.0002 (9)	0.0058 (9)	−0.0001 (10)
C7	0.0337 (11)	0.0421 (13)	0.0610 (16)	0.0041 (10)	0.0038 (10)	0.0111 (12)
C8	0.0279 (10)	0.0437 (13)	0.0449 (12)	0.0028 (9)	0.0057 (9)	−0.0062 (10)
C9	0.0505 (15)	0.0572 (17)	0.0653 (17)	0.0003 (13)	0.0173 (13)	−0.0208 (14)
C10	0.0335 (12)	0.0614 (17)	0.0661 (17)	−0.0081 (12)	0.0118 (11)	−0.0125 (14)

### Geometric parameters (Å, °)

**Table:**

O1—C1	1.316 (2)	C2—H2A	0.9900
O2—C1	1.225 (2)	C2—H2B	0.9900
C1—C2	1.502 (3)	C3—H3A	0.9900
C2—C3	1.523 (3)	C3—H3B	0.9900
C3—C4	1.498 (3)	C5—H5A	0.9900
C4—O3	1.211 (2)	C5—H5B	0.9900
C4—O4	1.338 (2)	C5—H5C	0.9900
O4—C5	1.449 (2)	C5—H5D	0.9900
C5—C6'	1.408 (16)	C6—H6	1.0000
C5—C6	1.492 (3)	C7—H7A	0.9900
O5—C6'	1.409 (17)	C7—H7B	0.9900
O5—C8	1.424 (3)	C7—H7C	0.9900
O5—C6	1.424 (3)	C7—H7D	0.9900
O6—C7	1.424 (3)	C6'—H6'	1.0000
O6—C8	1.424 (3)	C9—H9A	0.9800
C6—C7	1.537 (3)	C9—H9B	0.9800
C7—C6'	1.629 (17)	C9—H9C	0.9800
C8—C9	1.505 (3)	C10—H10A	0.9800
C8—C10	1.505 (3)	C10—H10B	0.9800
O1—H01	0.80 (3)	C10—H10C	0.9800
			
O2—C1—O1	123.58 (17)	C6'—C5—H5B	122.2
O2—C1—C2	122.95 (17)	O4—C5—H5B	110.3
O1—C1—C2	113.42 (16)	C6—C5—H5B	110.3
C1—C2—C3	113.37 (16)	H5A—C5—H5B	108.5
C4—C3—C2	112.54 (16)	C6'—C5—H5C	106.1
O3—C4—O4	123.59 (18)	O4—C5—H5C	106.1
O3—C4—C3	125.36 (18)	H5A—C5—H5C	137.6
O4—C4—C3	111.05 (16)	C6'—C5—H5D	106.1
C4—O4—C5	117.01 (16)	O4—C5—H5D	106.1
C6'—C5—O4	124.8 (7)	H5C—C5—H5D	106.3
O4—C5—C6	107.25 (17)	O5—C6—H6	110.3
C6'—O5—C8	102.9 (7)	C5—C6—H6	110.3
C8—O5—C6	106.90 (17)	C7—C6—H6	110.3
C7—O6—C8	106.95 (17)	O6—C7—H7A	110.9
O5—C6—C5	109.58 (19)	C6—C7—H7A	110.9
O5—C6—C7	104.30 (18)	O6—C7—H7B	110.9
C5—C6—C7	112.0 (2)	C6—C7—H7B	110.9
O6—C7—C6	104.48 (19)	H7A—C7—H7B	108.9
O6—C7—C6'	86.3 (7)	O6—C7—H7C	114.3
C5—C6'—O5	115.5 (14)	C6—C7—H7C	77.1
C5—C6'—C7	111.3 (13)	C6'—C7—H7C	114.3
O5—C6'—C7	100.5 (11)	O6—C7—H7D	114.3
O6—C8—O5	104.06 (17)	C6—C7—H7D	130.1
O6—C8—C9	111.3 (2)	C6'—C7—H7D	114.3
O5—C8—C9	110.8 (2)	H7C—C7—H7D	111.4
O6—C8—C10	109.0 (2)	C5—C6'—H6'	109.7
O5—C8—C10	108.9 (2)	O5—C6'—H6'	109.7
C9—C8—C10	112.4 (2)	C7—C6'—H6'	109.7
C1—O1—H01	109 (2)	C8—C9—H9A	109.5
C1—C2—H2A	108.9	C8—C9—H9B	109.5
C3—C2—H2A	108.9	H9A—C9—H9B	109.5
C1—C2—H2B	108.9	C8—C9—H9C	109.5
C3—C2—H2B	108.9	H9A—C9—H9C	109.5
H2A—C2—H2B	107.7	H9B—C9—H9C	109.5
C4—C3—H3A	109.1	C8—C10—H10A	109.5
C2—C3—H3A	109.1	C8—C10—H10B	109.5
C4—C3—H3B	109.1	H10A—C10—H10B	109.5
C2—C3—H3B	109.1	C8—C10—H10C	109.5
H3A—C3—H3B	107.8	H10A—C10—H10C	109.5
O4—C5—H5A	110.3	H10B—C10—H10C	109.5
C6—C5—H5A	110.3		
			
O2—C1—C2—C3	30.0 (3)	C5—C6—C7—C6'	55.6 (9)
O1—C1—C2—C3	−152.49 (16)	O4—C5—C6'—O5	18 (2)
C1—C2—C3—C4	67.0 (2)	C6—C5—C6'—O5	−57.7 (14)
C2—C3—C4—O3	−12.7 (3)	O4—C5—C6'—C7	131.5 (8)
C2—C3—C4—O4	167.00 (15)	C6—C5—C6'—C7	56.1 (12)
O3—C4—O4—C5	−8.3 (3)	C8—O5—C6'—C5	160.9 (13)
C3—C4—O4—C5	172.00 (16)	C6—O5—C6'—C5	59.9 (15)
C4—O4—C5—C6'	116.4 (12)	C8—O5—C6'—C7	41.0 (13)
C4—O4—C5—C6	157.92 (17)	C6—O5—C6'—C7	−60.0 (10)
C6'—O5—C6—C5	−51.5 (9)	O6—C7—C6'—C5	179.6 (14)
C8—O5—C6—C5	−142.17 (19)	C6—C7—C6'—C5	−60.5 (13)
C6'—O5—C6—C7	68.5 (9)	O6—C7—C6'—O5	−57.5 (11)
C8—O5—C6—C7	−22.1 (2)	C6—C7—C6'—O5	62.4 (11)
C6'—C5—C6—O5	53.2 (10)	C7—O6—C8—O5	−35.5 (2)
O4—C5—C6—O5	−70.5 (2)	C7—O6—C8—C9	83.9 (2)
C6'—C5—C6—C7	−62.0 (10)	C7—O6—C8—C10	−151.6 (2)
O4—C5—C6—C7	174.27 (18)	C6'—O5—C8—O6	−7.5 (11)
C8—O6—C7—C6	21.4 (3)	C6—O5—C8—O6	35.9 (2)
C8—O6—C7—C6'	54.2 (8)	C6'—O5—C8—C9	−127.2 (11)
O5—C6—C7—O6	0.5 (3)	C6—O5—C8—C9	−83.9 (2)
C5—C6—C7—O6	118.9 (2)	C6'—O5—C8—C10	108.6 (11)
O5—C6—C7—C6'	−62.8 (9)	C6—O5—C8—C10	152.0 (2)

### Hydrogen-bond geometry (Å, °)

**Table:**

*D*—H···*A*	*D*—H	H···*A*	*D*···*A*	*D*—H···*A*
O1—H01···O2^i^	0.80 (3)	1.86 (3)	2.6582 (19)	175 (3)
C3—H3A···O3^ii^	0.99	2.36	3.211 (2)	144
C2—H2B···O4^iii^	0.99	2.57	3.489 (2)	155
C7—H7B···O5^iv^	0.99	2.60	3.506 (3)	152

Symmetry codes: (i) −*x*+1, −*y*, −*z*+1; (ii) *x*, −*y*+1/2, *z*+1/2; (iii) *x*, *y*−1, *z*; (iv) *x*, −*y*+3/2, *z*−1/2.
